# Our Bureau of Information

**Published:** 1911-12-09

**Authors:** 


### OUR BUREAU OF INFORMATION.

Every week shows more clearly that the establishment of our Bureau was needed and is appreciated. We look forward to a steadily increasing usefulness for it. The average person who desires to help a sick or broken-down fellow-creature in whom he is interested knows at most but a limited number of institutions, none of which may exactly meet his wants, while the very place he is looking for may be available if he only knew of it. Through our Bureau he has every chance of finding out. Already numerous replies have been received for some of those who have asked our help; the inquirers have a much greater choice than they would have had without us. That is our object. As our list of approved homes grows it may be even simpler to choose an institution, as our representatives and correspondents will keep us informed of the special merits of different places. It is needless to say that all information given by our correspondents will be kept sacredly private and confidential.

### Correspondents and Helpers.

Certain points are obviously necessary in accredited correspondents. (1) They must have genuine experience in the needs of the sick, whether gained in private life or as a district visitor, district nurse, hospital worker, or any other position which brings them into practical touch with the sick and institutions which care for them. It is obvious that we cannot do justice to those who ask our advice if we are not sure that those who give us information are not only conscientious but well-informed. We, therefore, intend no discourtesy to any who wish to help us if in acknowledging their offer of assistance we ask them to furnish credentials of their fitness for what is a true and practical form of charity, and is also a responsible task. (2) We cannot accept as a correspondent anyone who has a personal or pecuniary interest in any home in the neighbourhood he wishes to represent. This is a work which must be purely disinterested.

### Coupons and Pseudonyms.

We must impress upon all who write to us either to ask for help or to offer homes that they must enclose the coupon to be found on the third page of the cover. No such communication will be noticed if it does not comply with this simple but essential condition. Many of our correspondents make it impossible for us to deal with their letters because they ask us not to publish their names, yet give us no pseudonym under which replies can be addressed to them. As we do not send postal replies, most people, we think, would prefer to be referred to under a *nom-de-plume*; but if they do not take this precaution, they must not complain if their name is given. We would, however, ask correspondents to avoid such names as "Reader" or "Constant Reader," which are likely to lead to confusion.

We should be grateful if inquirers whose needs have been met through our Bureau will inform us of the fact, as this saves forwarding unnecessary replies. A moment's thought will show them how desirable this is, both for the editor and for those who reply to the inquiries. We have a large number of letters to forward to an inquirer whose needs may be already met. If he will at once inform us of this fact we shall announce the fact in our next issue, and thus stop further communications coming. Where no such notice is given we must assume that the want is not met, and go on forwarding any letters that are sent to us.

We again invite impartial correspondents to tell us of homes or institutions in their neighbourhood which are suited for special cases, even though no inquiries regarding such cases may have yet appeared in our columns. To have such knowledge at our command will enable us to deal the more promptly with cases brought to our notice. And, be it remembered, we need and wish for information from every quarter of the British Isles. We need information about all sorts of institutions, about purely charitable ones, about those in which payments are asked for on a remunerative basis, and on all between. There is a special demand from people of moderate income for homes where those they are interested in can be taken at sums which cover, or little more than cover, the cost of maintenance. There are many such homes, but they are unknown to our inquirers. A list of these, in every part of the kingdom, would be a boon to many.

### How to Make the Bureau a Success.

Everyone who uses or wishes to help our Bureau of Information must be kind enough to observe the following rules:

(1) Postal replies cannot be given. Exception will only be made in very special cases at the Editor's discretion.

(2) Queries or answers relating to different cases must be written, or preferably typed, on separate sheets of paper, and the writing must never be crossed.

(3) Questions and replies received not later than Wednesday will, as far as possible, be dealt with in our issue of the following Saturday week.

(4) Letters must have attached the name and address of the sender, with a pseudonym for publication if preferred.

(5) *All communications* for this department *must* be *accompanied by the coupon* to be found at the bottom of the third page of the cover on the inside, and should be addressed to the Editor of the Bureau of Information, c/o The Hospital, 29 Southampton Street, Strand, London, W.C.

*Notice.*—We invite workers of special experience and knowledge to offer help in replying to questions relating to their special department.

### Needs and Helpers.

"Forward" writes: I am most anxious to know what kind of sputum-destructor has been found the most efficient for dealing with the sputum of consumptive patients, at what sanatorium it is in use, what firms supply it, and what is the cost?

Coupons not enclosed by M. P., Bedford; Sister-in-Charge, Abbey Road, Oxford; Sister Rideout; "Spanton"; Walpole Road; P. Beecroft.

Testimonials not enclosed by "Clactonian," "Rita," Wisbech (no pseudonym), Nurse Martin, Nurse Dora, Nurse Channell, Sister Mildred.

The following are thanked for their communications: Sister Faith, "North Hants," "Nil Desperandum," F. W. Hooper, Nurse A. Simmons, Burgoyne.

### PAYMENT FOR MEDICAL ATTENDANCE IN A HOSPITAL.

"M.D." writes from Fife as follows: Would you kindly let me know if it is a usual condition for patients to go into an ordinary hospital, and the doctors be able to charge for work done there, surgical and medical? Do you know of any hospital where this is done?

[It is not usual for a patient to go into an ordinary hospital and be charged a fee by the member of the medical staff who attends him. In a few instances up and down the country there are hospitals which have provided wards where patients are admitted on payment, and where, in the smaller hospitals, we believe, the patients do make arrangements for a payment to the attending practitioner. We shall be glad to have full information from those hospitals where some such practice exists at the present time.]

